# Innovative fakultative Seminarkonzepte besonders klinisch-praktisch ausgerichteter Lehre zur Famulatur- und PJ-Vorbereitung aus spezifisch chirurgischer Sicht

**DOI:** 10.1007/s00104-022-01757-x

**Published:** 2022-11-23

**Authors:** Philipp Stieger, Claus Schildberg, Marc Gottschalk, Katrin Werwick, Jonathan Hunger, Felix Walcher, Frank Meyer, Christian Albert, Rüdiger C. Braun-Dullaeus

**Affiliations:** 1 Universitätsklinik für Kardiologie und Angiologie, Universitätsklinikum Magdeburg A. ö. R., Magdeburg, Deutschland; 2 Universitätsklinik für Allgemein- und Viszeralchirurgie, Universitätsklinikum der MHB im Verbund Brandenburg an der Havel, Brandenburg an der Havel, Deutschland; 3 Studiendekanat der Medizinischen Fakultät, Otto-von-Guericke-Universität zu Magdeburg, Magdeburg, Deutschland; 4 Klinik für Neurologie, Klinikum Magdeburg GmbH, Magdeburg, Deutschland; 5 Universitätsklinik für Unfallchirurgie, Universitätsklinikum Magdeburg A. ö. R., Magdeburg, Deutschland; 6 Universitätsklinik für Allgemein-, Viszeral-, Gefäß- und Transplantationschirurgie, Universitätsklinikum Magdeburg A. ö. R., Leipziger Str. 44, 39120 Magdeburg, Deutschland

**Keywords:** Praxisbezogene Lehrinhalte, Interdisziplinarität, Interprofessionalität, Intersektorale Lehre, Praxisphasenvorbereitende Seminarreihe, Practice-related teaching content, Interdisciplinary concepts, Interprofessional collaboration, Intersectoral teaching, Seminar series in preparation for the practical phase

## Abstract

**Hintergrund:**

Die großen Praxisphasen der Famulaturen und des Praktischen Jahres (PJ) nehmen für Medizinstudierende eine besondere Stellung innerhalb ihres Kurrikulums ein.

**Ziel:**

Bezüglich Famulatur und PJ in der medizinischen Ausbildung wird exemplarisch ein Konzept zu vorbereitenden Seminarreihen einschließlich initialer Praxiserfahrungen vorgestellt.

**Methode:**

Es wird eine narrative Übersicht gegeben.

**Ergebnisse:**

Als gemeinsames Ziel der fakultativ initiierten Lehrveranstaltungen sollen die Studierenden zum Absolvieren von Famulatur und PJ besser qualifiziert und befähigt werden sowie das empfundene Zutrauen der Studierenden substanziell erhöhen. Die Erfahrungen in Famulaturen und PJ prägen Interesse und die Entscheidungen für ein Fach und den weiteren ärztlichen Berufsweg. Die Inhalte der hier vorgestellten Seminare zur Vorbereitung der ersten Famulatur und des ersten PJ-Tertials leisten einen Beitrag zur späteren selbstständigen ärztlichen Tätigkeit. Sie sollen vor dem folgenden Berufsstart für das Konzept einer umfassenden, d.h. komplexen interdisziplinären, -professionellen und -sektoralen Patientenversorgung sensibilisieren. Unter Berücksichtigung der jeweils unterschiedlichen Vorerfahrungen aus vorangegangenen Praktikumsabschnitten werden die Studierenden gezielt zu breit gefächerten, chirurgisch-interdisziplinären Lernzielen einer „Versorgungskompetenz“ unterrichtet. Studierende sollen auf diese Weise von den anregungsreich zu gestaltenden Phasen der Famulatur und des PJs vermehrt profitieren.

**Schlussfolgerung:**

Von einer Verbesserung der Lehre durch eine statusgerechte Vorbereitung, die neben typischen Tätigkeiten Studierender des Praktikums auch unmittelbar auf den ärztlichen Alltag vorbereitet, sind ein größerer Lernerfolg und ein verbessertes Praktikumserleben zu erwarten.

## Abstract (en)

**Background:**

Practice-oriented phases, such as the mandatory clinical traineeships and the final clinical internship, are of great importance in the teaching curriculum and skilful learning of medical students.

**Aim:**

With respect to the practical phases, such as clinical clerkship and medical internship, the concept of two innovative courses to prepare and evaluate these crucial training sections is presented including initial experiences from teaching practice.

**Method:**

A narrative review is given.

**Results:**

A common aim of facultatively initiated lectures is a better qualification of medical students to fulfil the requirements of clerkship and the last practical year of the study of human medicine to facilitate taking first steps towards professional work as a clinical physician, in particular, the self-confidence of the medical students is to be substantially increased. The experiences obtained during clerkship and the last practical year influence interest, motivation and final choice for a certain medical speciality. In that respect, this period is of great importance for the whole professional career. The content of the preparation courses for the first medical clerkships and the final clinical internship provide a valuable contribution to prepare for challenging clinical work as a physician with sole responsibility. In particular, they aim to introduce students to the concept of a multiprofessional and extensive patient care. Taking into account the different practical experiences obtained in previous study sections, students are taught according to the overall aim to achieve an interdisciplinary competence in clinical care.

**Conclusion:**

The improvement of teaching and optimized preparation for practical phases in medical studies promotes a more successful learning process during the clerkship and last practical year.

## Einleitung

Im Humanmedizinstudium sind im Rahmen der deutschen Ärztlichen Approbationsordnung (ÄApprO) neben dem Krankenpflegedienst insbesondere die Famulatur und das „Praktische Jahr“ (PJ) als große, zusammenhängende Praxisphasen im ärztlichen Arbeitsbereich vorgesehen [1]. Mit Beginn der jeweiligen Praktika ist gleichermaßen der Übergang in eine neue Ausbildungsphase verbunden, die als Statuspassage aufgefasst werden kann [2, 3]. Darüber hinaus haben die Praxisphasen für die Studierenden noch eine weitere wichtige Bedeutung. Erfahrungen, die in den intensiven, aber zeitlich eng umschriebenen Praktikumsphasen gemacht werden, haben prägenden Charakter für den künftigen Weg der Studierenden [4]. Aus didaktischer Sicht sollte somit eine fokussierte Betrachtung der Lehre in den Praxisphasen des Medizinstudiums erfolgen [5, 6].

Für das chirurgische Fachgebiet mehren sich Publikationen, die sich mit der Lehre in den Praxisphasen aus chirurgischer Perspektive befassen und dabei unterschiedlichste Bemühungen und innovative Ansätze aus dem chirurgischen Fachgebiet zur Verbesserung der Lehre dokumentieren [4, 7–13].

### Ziel.

Gegenstand der vorliegenden Ausführung ist die exemplarische Darstellung eines rahmenden Ausbildungskonzepts mit Inhalten zur Vorbereitung auf eine trotz studentischem Status stark bereits ärztlich geprägte Mitarbeit in der ersten Famulatur und vor Beginn des ersten PJ-Tertials. In hierzu konzipierten Blockseminaren sollen Studierende statusgerecht auf die Aufgaben der ärztlichen Mitarbeit in der PatientInnenversorgung sensibilisiert, instruiert und praxiswirksam bzw. -nah vorbereitet werden. Hierzu trainieren die Teilnehmenden interdisziplinäre, -professionelle und -sektorale Aufgabenstellungen der stationären PatientInnenversorgung. Anhand exemplarischer Krankheitsfälle einer Fallvignette lernen die Studierenden Grundprinzipien individuell ausgerichteter Behandlungskonzepte und den Organisationsprozess hinter der Krankenversorgung unter Einbeziehung interprofessioneller Expertise kennen. Hierzu werden Übungen der Kurven- und Visitenführung, der angemessenen Artikulation, zu Aspekten der Selbstorganisation sowie der Übergabe bis hin zur Gesprächsführung durchgeführt. Zur Betonung der Notwendigkeit interdisziplinärer Zusammenarbeit üben Studierende u. a. ganz konkret das Formulieren von Konsilanfragen sowie deren Dokumentation. Durch Unterrichtseinheiten zur Bedeutung des Arztbriefes, Anordnung von Physiotherapie bzw. Rehabilitationsmaßnahmen, Verordnungen etc. werden in theoretischen und praktischen Einheiten intersektorale Aspekte der PatientInnenversorgung unterrichtet und trainiert. Auf diese Weise erfahren Inhalte der Praxisphasen vom Zeitpunkt der Famulatur bis zum PJ eine begleitende Lehre und kurrikulare Rahmung, ohne die Eigenständigkeit der Ausführung der „laufenden“ Lehre der ausbildenden Kliniken und Stationen zu beeinträchtigen. Durch einen erleichterten Eintritt in die Praxisphasen als Folge einer zielgerichteten Vorbereitung soll überdies zu einem verbesserten Lernerfolg in den didaktisch wichtigen Praxisphasen beigetragen werden.

## Eckpunkte

### Etablierte Praxisphasen im Medizinstudium vs. Innovationsbedarf in der Lehre

#### Stellenwert der Famulatur in der ärztlichen Ausbildung

Im Rahmen der Famulaturen kommt es während insgesamt 120 Kalendertagen ganztägiger Praktikumsarbeit in

- einmal stationär,
- einmal ambulant,
- einmal hausärztlich (und)
- einmal frei wählbar

zu den ersten Einsätzen im klinischen Alltag auf ärztlicher Seite (§ 7 Abs. 2 ÄApprO). Die Studierenden sollen in der Famulatur „mit der ärztlichen PatientInnenversorgung in Einrichtungen der ambulanten und stationären Krankenversorgung vertraut“ gemacht werden [1]. Während für die Ausbildung im Rahmen der Famulatur bereits verschiedene Berichte über erfolgreiche Kurrikula und Strukturierungen existieren, gibt es nach Wissen der AutorInnen bis heute keine Ansätze, die die ärztlichen Praxisphasen kurrikular verbinden [1–3]. Die Inhalte der Famulaturen unterliegen formal nicht der Verantwortung und Strukturierung bzw. den Vorgaben der Medizinischen Fakultäten. Der Lernerfolg eines Praktikums und insbesondere die Verknüpfung (universitäts-)theoretischer Inhalte mit dem Erleben ihrer klinisch-praktischen Anwendung ist jedoch ein nicht unerheblicher Schritt auf dem Weg Medizinstudierender in den späteren klinischen Berufsalltag [4]. Es ist daher für den Lernerfolg des jeweiligen Praktikums und mit Hinblick auf das Ziel ärztlicher Ausbildung eines Berufsstarts in der PatientInnenversorgung besonders wichtig, Praxiserlebnisse strukturiert zu reflektieren.

Als Schwierigkeit fakultätsferner Ausbildung in Famulaturen an unterschiedlichsten Einrichtungen ergibt sich, dass die Rolle und das Aufgabenspektrum der/des Famulierenden^1^ bisher weder wissenschaftlich noch didaktisch erschöpfend ausdifferenziert ist! Obwohl als Pflichtteile des Medizinstudiums ähnlich den heutigen Bedingungen bereits seit über 70 Jahren etabliert, werden Inhalte und Reflexionen der Tätigkeiten Studierender im komplexen Zusammenwirken des ärztlichen Alltags in der Rolle als „randständiger Novize“ in medizinischen Praktika bisher nicht systematisch mit dem medizinischen Kurrikulum verknüpft [5, 6].

Das legt die systematisierende Eingliederung derartig kreierter Ausbildungsinhalte ins praxisrelevante Humanmedizinstudium nahe, dem nicht zuletzt auch diese Übersicht gerecht werden will.

Die Famulatur fordert von den Studierenden die Anwendung von

- Wissen,
- Fertigkeiten (und)
- professionellen Haltungen in konkreten Situationen [7].

Die Studierenden müssen aktiv einen komplexen Theorie-Praxis-Transfer bewältigen und werden gleichzeitig in die medizinische Berufswelt sozialisiert [8].

Auch erfahren Studierende die Notwendigkeit aktiver interprofessioneller und -disziplinärer Zusammenarbeit in der alltäglichen PatientInnenversorgung des klinischen Betriebes. Diese möglichst frühzeitige Kooperation in der klinischen Tätigkeit ist von Beginn der ärztlichen Praktika an von großer Bedeutung für die Studierenden und kann im Falle erfolgreicher Durchführung prägend für die ersten fachlichen Weiterbildungspräferenzen sein [4, 9, 10]. Bis dahin lernen die Studierenden in keinem anderen Ausbildungsabschnitt das gewählte Fach so intensiv wie in der Famulatur kennen. Hierbei legen Studierende besonderen Wert auf das Erlernen praxisrelevanter Tätigkeiten, die mehrheitlich auf eine aktive Mitarbeit ausgerichtet sind. In der Vorstellung einer „Schüler-Mentor-Beziehung“ erwarten Studierende eine gute Betreuung und Einbindung in die klinischen Arbeitsweisen [11]. Sofern eine effektive Integration in die Versorgungsabläufe möglich ist, bietet die Famulatur die besondere Chance, Studierende früh für ein Fach zu begeistern, ihnen mögliche spätere Tätigkeitsfelder näher zu bringen und ggf. auch an die Chirurgie zu binden [4].

#### Stellenwert des „Praktischen Jahres“ in der ärztlichen Ausbildung

Vor Ende des Medizinstudiums und dem dritten Abschnitt der ärztlichen Prüfung ist mit den Pflichtfächern Chirurgie und Innere Medizin sowie Wahlbereichen und -fächern das PJ abzuleisten. Im PJ ist durch § 3 der ÄApprO festgelegt, dass die Studierenden die „im Studium erworbenen ärztlichen Kenntnisse, Fähigkeiten und Fertigkeiten vertiefen und erweitern sollen. Sie sollen lernen, sie auf den einzelnen Krankheitsfall anzuwenden“ [12]. Unmittelbar vor Abschluss des universitären Teils der Ausbildung soll im Übergang vom Hochschulstudium in das ärztliche Arbeitsfeld das PJ den Praxisbezug der Ausbildung vervollständigen und damit die AbsolventInnen auf einen anschließenden Berufsbeginn in der praktischen PatienteInnnversorgung vorbereiten. Die PJ-Studierenden können auf ihre Erfahrungen in den vorangegangenen praktischen Ausbildungseinheiten zurückgreifen [7]. Mit dem „Logbuch zum PJ“ erstellen die Universität bzw. die einzelnen FachvertreterInnen für alle Fachbereiche ein Kompendium von Lehrinhalten und Lernzielen, die den Studierenden im PJ vermittelt werden sollen. Für die Pflichttertiale Innere Medizin und Chirurgie liegen Basislogbücher des Medizinischen Fakultätentages (MFT) vor, die teils noch weiter durch einzelne Fakultäten in einrichtungsspezifischen Versionen konkretisiert wurden, wie in Magdeburg erfolgreich für die chirurgischen Fächer erfolgt und praxiswirksam etabliert^2^ [13–16].

Mit diesen Dokumentationsvorlagen sollen die Erarbeitung und Aneignung der jeweiligen fachlichen Hintergründe und damit verbundenen praktischen Fertigkeiten der ärztlichen Arbeit sichergestellt werden. Über manuelle Fertigkeiten hinaus besteht in der letzten Praxisphase vor Beginn der eigenständigen ärztlichen Tätigkeit eine Chance zum Erlernen und Anwenden kontextuellen Arbeitens in der PatientInnenversorgung. Dies beinhaltet u. a. das eigenständige Erstellen von Therapiekonzepten, das die Zusammenarbeit aller an der Versorgung Beteiligten im stationären Umfeld integriert. Für einen optimalen Lernerfolg im PJ wird folgende Strukturierung des Lernens favorisiert:

- studierendenzentriertes Lernen,
- Feedback,
- die Stimulation komplexer Denkvorgänge, aber auch
- patientenorientierte Empathie (und)
- Wertschätzung [7, 17, 18].

#### Innovative Lehransätze

Hierzu werden zwei unterschiedliche Seminare vorgestellt, in denen modular aufgebaute Unterrichtseinheiten in fokussierten Blockseminaren (PJ) auf die jeweils anstehenden Praktika vorbereiten. Sowohl zur Famulatur als auch zum PJ werden medizinisch-praktische und -theoretische Inhalte aus dem ärztlichen Alltag der jeweiligen Fachgebiete aufgefrischt bzw. interdisziplinäre, -professionelle und -sektorale Aspekte der Aufgabenstellung durch entsprechende fachspezifische DozentInnen unterrichtet. Studierenden soll hierdurch in chirurgischen und nichtchirurgischen (z. B. internistischen) Praxisphasen und den damit verbundenen Statuspassagen zu einem verbesserten Erleben der Praktika und der Fachgebiete verholfen werden. Davon verspricht man sich eine bessere Identifikation mit der klinischen Medizin im Allgemeinen und insbesondere mit dem (jeweils) präsentierenden klinischen Fach.

Durch das Angebot der hier vorgestellten Seminare kann ein Beitrag zur wichtigen Aufgabe eines praxisphasenbegleitenden Unterrichts kurrikular geleistet werden.

Zur Vermittlung chirurgischer und internistischer Schwerpunktthemen typischer studentischer Tätigkeiten im Praxisalltag, aber auch der Darstellung der jeweiligen fachspezifischen Lösungsstrategien klinischer Fragestellungen wurden Vorbereitungsseminare zur ersten Famulatur und zum Beginn des PJ jährlich in mehreren aufeinanderfolgenden Wintersemestern an der Medizinischen Fakultät Magdeburg durchgeführt. Anhand einer zugrunde liegenden, sich inhaltlich entwickelnden Fallvignette wurden interdisziplinäre, -professionelle und -sektorale Aspekte der klinischen Aufgaben in der PatientInnenversorgung erörtert und in theoretischen und praktischen Einheiten in chirurgischen und internistischen Modulen der jeweiligen Blockseminare trainiert.

Durch die Übertragung der Erfahrungen und Ergebnisse der durchgeführten interdisziplinären Seminare zur Vorbereitung auf die erste Famulatur und den Beginn des PJ kann ein effektiver Beitrag zur

- Nachwuchsrekrutierung insbesondere in chirurgischen Fächern [4],
- Zufriedenheit der Studierenden (Daten unveröffentlicht) und
- selbsteingeschätzten Verbesserung des Lernerfolgs in der jeweiligen Praxisphase [6]

geleistet werden.

### Praxisphasen mit klinisch(-chirurgisch)er Kompetenzorientierung

Künftige Ärztinnen und Ärzte sollen durch Absolvieren der Praxisphasen an die praktische Medizin herangeführt werden mit dem Ziel, eine professionelle Handlungskompetenz für den Berufseinstieg in der Patientenversorgung zu erwerben (vgl. § 7 Ärztliche Approbationsordnung [ÄApprO]). Zum Erlangen einer Kompetenz, die zur ärztlichen Versorgung eines individuellen PatientInnenfalles befähigt, ist jedoch mehr als Wissen erforderlich. Nach der Definition von Weinert sind Kompetenzen grundsätzlich „die bei Individuen verfügbaren oder durch sie erlernbaren kognitiven Fähigkeiten und Fertigkeiten, um bestimmte Probleme zu lösen, sowie die damit verbundenen motivationalen, volitionalen und sozialen Bereitschaften und Fähigkeiten, um die Problemlösung in variablen Situationen erfolgreich und verantwortungsvoll nutzen zu können“ [19]. Die Vermittlung von Kompetenzen erfordert daher im Vergleich zu reiner Wissensvermittlung eigene Unterrichtsformen und Methoden, die in hohem Maße durch Anwendung und Erleben „am Ort des Geschehens“ profitieren. Um der Komplexität von Gesundheit und der Entstehung von Krankheit Rechnung zu tragen, soll eine Vermittlung von kompetenzorientierten Lernzielen vor allem fächerübergreifend erfolgen [20]. Diese Vorgaben werden durch die hier vorgestellten Vorbereitungsseminare auf die Praxisphasen in besonderem Maße erfüllt. In Anlehnung an den Nationalen Kompetenzbasierten Lernzielkatalog Medizin (NKLM) können unterschiedliche Schritte zur Erlangung einer Handlungskompetenz in eine Sequenz gegliedert werden: Durch die

- Aneignung von Faktenwissen (Was?) und
- Handlungs- und Begründungswissen (Wie und Warum?)

ist zunächst die Grundlage einer Handlungskompetenz geschaffen. Über eine aktive, angeleitete Anwendung von Lerninhalten werden dann eigene Erfahrungen gewonnen [21]. Als „anvertraubare professionelle Tätigkeiten“ (APT) bestehen vergleichbare Konzepte für eine kompetenzbasierte Ausbildung im PJ und in der postgraduierten Weiterbildung [22, 23]. APT definieren eine reale professionelle Handlung in einem definierten Kontext, die an die/den Lernende(n) übertragen werden kann. Die Ausführung der APT beruht auf mehreren Kompetenzen, die für diese professionellen Handlungen wichtig sind. In zunehmender Selbständigkeit werden Lernende schließlich in den spezifischen Tätigkeiten unter abnehmendem Supervisionsgrad strukturiert geschult. Die ÄApprO beschrieb das Ziel der Ausbildung im PJ: „Zu diesem Zweck sollen die PJ-Studierenden entsprechend ihrem Ausbildungsstand unter Anleitung, Aufsicht und in Verantwortung der/des ausbildenden Ärztin/Arztes ihnen zugewiesene ärztliche Verrichtungen durchführen“ (§ 3 Abs. 4 ÄApprO). In der grundsätzlichen Betrachtung beider Praxisphasen lassen sich Famulatur und PJ als aufeinander aufbauend sowohl mit Hinblick der Reihenfolge im Studium als auch der Teilnahme an ärztlichen Tätigkeitsbereichen unterscheiden. Naheliegend und didaktisch zu nutzen ist eine kurrikulare Rahmung durch Schwerpunkte, die gleichermaßen in den Praxisphasen Famulatur und PJ aufeinander aufbauen.

### „Versorgungskompetenz“ – der kurrikulare Rahmen für Famulatur und PJ

Innerhalb der letzten Jahre hat die kompetenzbasierte medizinische Ausbildung zunehmend an Einfluss und Bedeutung in der praxisorientierten humanmedizinischen Lehre gewonnen. Als Besonderheit der kompetenzbasierten Ausbildung kann u. a. gezielt ein Fokus auf spezifische Kompetenzbereiche gesetzt werden, die unabhängig vom Zeitpunkt innerhalb sowohl der Aus- als auch Weiterbildung anwendbar sind [24].

Obgleich in der ÄApprO § 7 als Lernziel des Medizinstudiums festgelegt und im praktischen Alltag unmittelbar anzuwenden, sind die Inhalte, die eine zusammenfassende Versorgung eines Krankheitsfalles betrachten, bisher kaum Gegenstand der praktischen Ausbildung im Medizinstudium. Daher ist es wichtig, die Rolle der Versorgungskompetenz in der studentischen Ausbildung zu stärken [8]. In einem übergeordneten Konzept kann die Vermittlung einer Versorgungskompetenz auf drei Teilaspekte didaktisch fokussiert und in den unterschiedlichen praxisanleitenden Phasen rahmend angewendet werden:

- interprofessionelle Zusammenarbeit,
- Erarbeiten eines individuellen Behandlungskonzepts durch Bezug des Einzelfalls auf das System (und)
- ein fachübergreifendes Verständnis medizinisch-therapeutischer Handlungsabläufe.

Praxisphasenvorbereitende Unterrichtseinheiten bieten mit Hinblick auf die bevorstehende Aufgabe zur Translation theoretischen Wissens in den praktischen Zusammenhang eine geeignete Gelegenheit, Schwerpunkte klinischer Versorgung zu verdeutlichen.

Als Beispiel für ein Konzept praxisphasenvorbereitender Unterrichtseinheiten mit Berücksichtigung von Lernzielen ärztlicher Versorgungskompetenz soll im Folgenden Bezug auf die Seminarreihe zur Vorbereitung der ersten Famulatur und dem PJ der Medizinischen Fakultät Magdeburg genommen werden. Sie setzt sich aus

- einem zweitägigen Blockseminar zur Vorbereitung auf die erste Famulatur und
- einem fünftägigen Blockseminar zur Vorbereitung auf das erste PJ-Tertial

im Rahmen eines umschriebenen Kurrikulums zur Versorgungskompetenz der Magdeburger Medizinischen Fakultät zusammen [8].

Ein wichtiges Element der frühen klinischen Lehre ist das Arbeiten mit Fallbezug [25]. Ein wesentlicher Bestandteil der Vorbereitungsseminare zu den Praxisphasen Famulatur und PJ ist daher die aktive Erarbeitung konkreter theoretischer und praktischer Aufgabenstellungen zu chirurgischen und nichtchirurgischen Fragestellungen einer begleitenden Fallschilderung.

### Fallbezogene Ausbildung in der Vorbereitung der Praxisphasen

Studierende sind in Praxisphasen häufig mit der Problematik konfrontiert, dass das „Wissen theoretisch abrufbar ist, aber unzureichend im (beruflichen) Handeln wirksam wird“ und sie gleichzeitig im praktischen Einsatz „unter einem erheblichen sozialen Bewährungsdruck“ stehen [26, 27]. Auf die mangelnde Einbettung medizinischen Sachwissens in einen für die/den Lernende(n) bedeutsamen Kontext reagieren viele Universitäten mit der Etablierung fallbasierter Lern- und Lehrkonzepte. Das Lernen am Beispiel authentischer Fälle „integriert die Vermittlung von Hintergrundwissen in praktische Problemstellungen“ und ermöglicht dabei die gemeinsame Erarbeitung neuer „Wissensstrukturen (…), auch wenn die verfügbare Wissensbasis eines Lernenden unvollständig ist“ [25, 28]. Diese Methodik beweist sich lern- und kognitionspsychologisch, insbesondere „in Bereichen, in denen die Probleme sehr komplex sind und/oder es keine Formalisierung gibt“, etwa bei der Erarbeitung medizinischer Diagnosen [28, 29].

Nicht zuletzt tragen die realitätsnahe und didaktisch reduzierte Aufbereitung der Fälle, an welche die Teilnehmenden häufig mit eigenen Erfahrungen aus dem klinischen Kontext anknüpfen können, die angstfreie Lernsituation im geschützten Rahmen einer Kleingruppe und der episodische Charakter der Fälle, „die wie spannende Geschichten erzählt werden (können)“, zu einer Motivationssteigerung bei den Lernenden bei [30]. Dies äußert sich auch in der positiven Bewertung entsprechender Lehrkonzepte durch die Studierenden [8].

Gängige Formate sind dabei

- das „problemorientierte Lernen“ (POL),
- Vorlesungen mit Fallbeispielen („lecture-based cases“) (oder)
- die Fallmethode („case method“), bei der ein exemplarischer Fall mit zugehöriger Lösung im Rahmen eines Seminars diskutiert wird [30, 31].

Allen geschilderten Ansätzen ist die Fokussierung auf didaktisch aufbereitete, typisierte Fälle gemein, anhand derer die Zusammenhänge medizinischer Befunderhebung, Diagnosestellung und daraus resultierenden Behandlungsoptionen erarbeitet und diskutiert werden sollen.

Neben praktischen Übungseinheiten und Reflexionen der Aufgaben ärztlicher Tätigkeitsbereiche kam dieser Ansatz als elementarer Bestandteil des didaktischen Konzepts in den Vorbereitungsseminarreihen zu den Praxisphasen Famulatur und PJ an der Medizinischen Fakultät Magdeburg zum Einsatz. Die seminarbegleitende Fallvignette war entsprechend der Tagesmodule (chirurgische oder nichtchirurgische Versorgung) anhand einer fiktiven PatientInnengeschichte entwickelt worden. Im Rahmen der einzelnen Stationen der PatientInnengeschichte sind Aufgabenstellungen interdisziplinärer, -professioneller und -sektoraler PatientInnenversorgung von der stationären Aufnahme des PatientInnenfalles bis zu dessen Entlassung für die Studierenden in theoretischen Erarbeitungen (z. B. Versorgungskette) und praktischen Übungen (Untersuchungstechniken, Formulieren von Konsilanfragen, Dokumentation in der PatientInnenkurve, Verfassen eines Arztbriefes etc.) nachvollziehbar geworden. Zu jeder Station der PatientInnengeschichte stellten sich den bearbeitenden Studierenden statusgerecht theoretische Überlegungen und Fragestellungen, die es erforderten, die nächsten Diagnose- und Behandlungsschritte abzuwägen und eine Reflexion der Zusammenhänge einer komplexen PatientInnenversorgung anzustellen. In begleitenden Praxiseinheiten wurden Kenntnisse und Fähigkeiten aufgefrischt, denen es zur Bearbeitung der Aufgabenstellungen bedarf. Sowohl bei der Bearbeitung der Fallvignette in Kleingruppen als auch den praktischen Übungen stand ein interprofessionelles bzw. interdisziplinäres Team aus Dozierenden zur Verfügung.

### Konzept der praxisphasenvorbereitenden Seminarreihe

Lernziele und Aufgabenstellungen der *praxisphasenvorbereitenden Seminarreihe* berücksichtigen den Status (Kenntnisstand, Erfahrungen, Erwartungen, Aufgaben im Praktikum, rechtlicher Rahmen der Tätigkeit etc.) der Studierenden und wurden nach deren typischen Einsatzbereichen der unterschiedlichen teilnehmenden Fächer ausgerichtet. Es wurden Lernziele unterrichtet, die neben fachlichen Schwerpunkten der jeweiligen Fächer unterschiedliche übergeordnete Lernziele und Aspekte/Dimensionen zur Stärkung der eigenen Rolle auf dem Weg zur selbständigen ärztlichen Tätigkeit fokussieren (Tab. 1).

**Table 1:** 

Studierende …	
*Vielfalt* können anhand einer Fallbearbeitung das klinische Spektrum von Basis- bis hochspezialisierter Versorgung darstellen entwerfen Versorgungsketten eines Falls mithilfe interdisziplinärer Kooperationen erfahren ambulante Betreuungsoptionen eines Fachs lernen anhand einer Fragestellung zur Versorgung neue Therapiemethoden des Fachs kennen (Roboterchirurgie, Hybridoperation etc.)	*Karrierechancen* lernen Einzelheiten möglicher Aus‑/Weiterbildungskurrikula durch MentorInnen kennen erfahren Aspekte der eigenen „Robustheit“ gegenüber den Anforderungen des Fachs erfahren unterschiedliche Aspekte („Höhen“ und „Tiefen“) einer Arbeit in einer chirurgischen Fachdisziplin bauen Barrieren hierarchischer Ebenen durch Kennenlernen von FachvertreterInnen im fachbezogenen Kleingruppenunterricht ab
*Praxisbezug* trainieren status- und rollenangepasste praktische Fähigkeiten für das anstehende Praktikum erarbeiten individualisiertes Fallmanagement trainieren den eigenen Aufgaben angepasste Notfallszenarien	*PatientInnenzentrierung* begegnen klinischer Alltagsrealität des jeweils gegebenen PatientInnenaufkommens reflektieren ethische Grenzsituationen eines Falles erleben klinisches Teamwork und konkrete Handlungsoptionen zur Versorgung eines PatientInnenfalles

#### Das Projekt „Fit für Famulatur“ (FfF)

In der vorlesungsfreien Zeit des Wintersemesters 2013/14 (*n* = 50 Teilnehmende) wurde an der Medizinischen Fakultät Magdeburg ein Pilotprojekt zu einem extrakurrikularen Seminar zur Vorbereitung auf die erste Famulatur („Fit für Famulatur“) initiiert und seither zum selben Zeitpunkt des jeweiligen Wintersemesters mit jeweils ähnlichen Teilnehmendenzahlen fortgesetzt. In einem zweitägigen Seminar (Tab. 2) erarbeiteten Studierende vor Antritt ihrer ersten Famulatur in einem internistischen und einem chirurgischen Modul (jeweils ein Tag mit eigener fachspezifischer Modulverantwortung) thematisch grundlegende Aspekte der stationären Versorgung im Hinblick auf ihre unmittelbar anstehende stationäre Tätigkeit als Famulierende. Inhaltlich sind beide Module durch eine rahmende Fallvignette einer fiktiven PatientInnengeschichte verbunden. In begleitenden Workshops zu praktischen Inhalten unterrichteten neben den ärztlichen Dozierenden u. a. examinierte MitarbeiterInnen der Krankenpflege, technische AssistentInnen, Mitarbeitende des Sozialdienstes sowie primärärztlich tätige KollegInnen klinische Skills zur Darstellung interprofessioneller bzw. -sektoraler Aspekte des Falls für den Tätigkeitsbereich einer/s Famulantin/Famulus. Zur Stärkung der Rolle der Famulierenden wurden ergänzende „Sessions“, wie z. B. ein

- Fachvortrag „Rechtlicher Rahmen Famulatur“,
- „Markt der (Famulatur‑)Möglichkeiten“ mit MentorInnengesprächen erfahrener Fachärztinnen/Fachärzte unterschiedlicher Disziplinen

**Table 2:** 

	Vormittag	Nachmittag	Zusatzangebot
*Tag 1*	Klinisch-internistische Versorgungskette – Bearbeitung der Fallvignette (Schwerpunkt: internistisch), Modulverantwortung: **Innere Medizin**	Skills-Parcours (Schwerpunkt: **internistisch**)	Fachvortrag „Rechtlicher Rahmen Famulatur“
*Tag 2*	Klinisch-chirurgische Versorgungskette – Bearbeitung der Fallvignette (Schwerpunkt: chirurgisch) Modulverantwortung: **Chirurgie**	Skills-Parcours (Schwerpunkt: **chirurgisch**) Operationssaalbesuch	Markt der Möglichkeiten (Kliniken und Lehrpraxen stellen sich vor) MentorInnengespräche fachärztlicher Vertreter unterschiedlicher Disziplinen

angeboten.

Fokus der jeweiligen Lehreinheiten ist die Einordnung des fiktiven medizinischen Falles einer älteren, multimorbiden Patientin in die typischen klinischen Versorgungsabläufe, an denen ein(e) Famulierende(r) im klinischen Alltag beteiligt ist. Die Studierenden lernen so unter ärztlicher und pflegerischer Anleitung Prozesse und Strukturen in der PatientInnenversorgung kennen (Integration in den Klinikalltag und interprofessionelle Teamarbeit im Fachbereich) und vernetzen diese mit ihrem theoretischen und praktischen Wissen.

Mit Blick auf die Chirurgie konnten den Studierenden weiterhin Basisfertigkeiten wie z. B. das Verhalten und die Strukturierung der Arbeitsabläufe im Operationssaal sowie Fertigkeiten wie chirurgische Händedesinfektion, Verbandslehre und Wunddokumentation sowie häufige Nahttechniken vermittelt bzw. aufgefrischt werden. In inhaltlich verbindenden Übungen sollten des Weiteren durch z. B. Formulieren von Versorgungsketten diagnostische Schritte und therapeutische Möglichkeiten interdisziplinärer Versorgungskonzepte anhand des in der Vignette vorgestellten Falles erarbeitet werden.

In Impulsreferaten wurden medizinisch-klinische Schlüsselschritte durch entsprechende FachvertreterInnen vorgestellt.

Es konnte gezeigt werden, dass Studierende die Vorbereitungsseminare als gute und praktisch relevante Vorbereitung auf die Famulatur wahrnehmen. Studierende haben nach der Veranstaltung eine bessere Vorstellung, wie sie sich in der Klinik einbringen können. Hierbei befürworten sie die vernetzte und interprofessionelle Wissensvermittlung [8].

#### Das Projekt „Fit für PJ“

Analog zu den famulaturvorbereitenden Seminaren wurden seit Wintersemester 2014/15 zu Beginn des Wintersemesters eine fakultative PJ-Vorbereitungsseminarwoche angeboten (initial: *n* = 120 TeilnehmerInnen). Als Erweiterung Skills-basierter Trainingskonzepte berücksichtigen die Konzepte der hier vorgestellten Vorbereitungsseminare die aktuelle Arbeitswirklichkeit auf Station als ärztlich Tätige in einem interprofessionellen Team, das Arbeitsabläufe im stationären Alltag strukturieren muss und anteilig organisiert. Hierzu bedarf es gesonderter administrativer Tätigkeiten, deren Kenntnisse z. B. hinsichtlich der Dokumentation der stationären Tätigkeit (PatientInnenakte, Konsilanforderungen etc.) für den Alltag wertvoll sind. An fünf Modultagen (Tab. 3) erarbeiteten Studierende vor Antritt ihres PJ thematisch grundlegende Aspekte der stationären Versorgung im Hinblick auf ihre unmittelbar anstehende Tätigkeit als PJ-Studierende. Analog zu „Fit für Famulatur“ wurden auch zur Vorbereitung auf das PJ in begleitenden Workshops praktische Inhalte der unmittelbar anstehenden Tätigkeiten interprofessionell und -disziplinär vermittelt. Zur Stärkung der Rolle der PJ-Studierenden wurden ergänzend Lehr- oder Unterrichtseinheiten, wie z. B. ein Fachvortrag „Rechtlicher Rahmen der Tätigkeit als PJ“ und „PJ-MentorInnen-Gespräche“ angeboten.

**Table 3:** 

	Vormittag	Nachmittag	
*Tag 1*	Notaufnahme	**Skills- und Workshop-Rotation** (Blutentnahme und Labormanagement, ärztliche Gesprächsführung, Ultraschall, „emergency communication, medical English“, postoperative Komplikation, dermatologische/allergologische Aspekte, Indikation und Anforderungen radiologischer Untersuchung, BLS bzw. ALS, Perfusor programmieren, Umgang mit i.v. Medikation, Verbandswechsel, Wundvisite, Nähen, Operationssaalbesuch, chirurgische Untersuchung Abdomen und Knie, medizinische Dokumentation und Arztberichterstattung, …)	Fachvortrag „Rechtlicher Rahmen PJ“, FachanwältIn für Medizinrecht/Diskussion
*Tag 2*	„Auf Station“ – Innere Medizin		PJ-MentoreInnen-Gespräche (fakultativ)
*Tag 3*	„Notfall auf Station und intensivmedizinische Versorgung“ (Anästhesie)		PJ-MentorInnen-Gespräche (fakultativ)
*Tag 4*	„Im Operationssaal – Chirurgie“		Arbeitsbereiche und Verhalten im Operationssaalbereich (Pflege)
*Tag 5*	Entlassung und ambulante Versorgung (Allgemeinmedizin)		Ambulante Medizin/poststationäre Versorgung aus Sicht von HausärztInnen

Der seminarbegleitende Fall führte die Teilnehmenden zur Vorbereitung auf die unterschiedlichen Tertiale und Fachbereiche im PJ in einem modularen Aufbau durch die einzelnen Etappen und Schritte der ärztlichen Versorgung eines Krankheitsfalles. Studierende lernen anhand einer Fallvignette unter einem täglichen Fachschwerpunkt zunächst Aufgaben und Anforderungen der Tätigkeit in einer Notaufnahmesituation (u. a. Anamnese, körperliche Untersuchung, Dokumentation, Anordnung, [Differenzial-]Diagnostik, Umgang/Kommunikation mit den MitarbeiterInnen der pflegerischen Ebene etc.).

Im folgenden Modul Innere Medizin betreuten Fachdozierende interprofessionell (Ärztinnen/Ärzte, Pflege, Physiotherapie, Sozialdienst) Anleitungen zur stationären Versorgung (Stationsalltag, Aufbau und Hintergrund einer Visite, Untersuchungsanmeldungen, Konsilanforderungen, Wundpflege/Verbandswechsel, Kommunikation mit den PflegemitarbeiterInnen, Dokumentation, medizintechnische Unterweisungen etc.; [32]).

Am dritten Tag, dem Modul Notfall/Intensivmedizin, wurde das Verhalten in einer Notfallsituation im stationären Alltag sowie Eckpfeiler der anschließenden intensivmedizinischen Versorgung statusgerecht erarbeitet.

Das vierte Modul fokussierte auf die chirurgische Betreuung inklusive Indikationsstellung, Algorithmus der spezifischen Diagnostik und schließlich die operative Versorgung des fiktiven Falles.

Abschließend und sektorenübergreifend wurde im letzten Modul die hausärztliche Weiterversorgung mit entsprechender Dokumentation („Bedeutung des Arztbriefes“, Übungen zum Arztbriefschreiben, Entlassungsmanagement etc.) von Dozierenden hausärztlicher akademischer Lehrpraxen vermittelt. Hierdurch sollen die Absolvierenden auf der Grundlage der im Studium erarbeiteten Kenntnisse und Fertigkeiten anhand exemplarischer Schwerpunkte typischer Tätigkeiten der jeweiligen Fachbereiche fokussiert unterrichtet und zum Erkennen und zur Übernahme statusangemessener Aufgaben als PJ-Studierende angeleitet werden.

Unter besonderer Beachtung des unmittelbar an das PJ anschließenden Berufsstarts werden über die Ausübung ärztlicher Rollen zudem Lernziele zur verbesserten Zusammenarbeit unterschiedlicher Berufsgruppen am Versorgungsauftrag trainiert (Stationsmanagement und Dokumentation, Hygiene und Infektionsmanagement, Sozialdienst; [33]).

### Spezifika – Chirurgie

Für die chirurgischen Fächer bieten praxisvorbereitende Seminare besondere Chancen. Durch gezielte Vorbereitung und damit Stärkung der studentischen Rolle im Praktikum kann der prägende Effekt klinischer Lehre sowohl zu Inhalten als auch dem Berufsfeld Chirurgie nachhaltig verstärkt werden [4]. Die vorgestellten Konzepte „Fit für Famulatur“ oder „Fit für PJ“ eignen sich dafür hervorragend. Sie erlauben, die Stärken des Faches den Studierenden nahezubringen und bei ihnen Neugier auf Famulatur und PJ zu wecken. Durch die vorbereitenden Seminare wird weiterhin die Vororteingewöhnung so kurz wie möglich gehalten, um so den maximalen Lehr‑/Lerneffekt dieser Praxisphasen zu steigern. Tab. 1 gibt einen Überblick über Lernziele der begleitenden Lehre von Praxisphasen, wie z. B. durch Lehre in Vorbereitungsseminaren zu Famulatur und PJ.

Die Chirurgie ist ein ausgezeichnet geeignetes klinisches Fach, um **i)** praxisorientierten Wissenstransfer zu realisieren, z. B. durch studierendenzentriertes Lernen, **ii)** adaptierte (kompetenzorientierte) Lernziele zu verfolgen, **iii)** praxisorientierte Lehre gut zu strukturieren (z. B. direkte Betreuung anvertrauter PatientInnen [u. a. „Handlungs‑/Versorgungskompetenz“]), **iv)** auf Fertigkeiten, patientenorientierte Empathie und eine professionelle Haltung zu orientieren,** v)** eine individuelle ärztliche Betreuung der/des FamulantIn/PJ-lerIn (1:1 [„SchülerIn-MentorIn-Beziehung“] oder Kleingruppe) zu gewähren, was nicht zuletzt **vi)** zeitnahe Rückkopplungen und stetig gelebte (Hinweise auf) **vii)** interdisziplinäre/-professionelle/-sektorale Zusammenarbeit erlaubt.

### Limitationen – Ausblick

Weiterführende Lehrkonzepte wie die fakultative Seminarreihe zu den Praxisphasen Famulatur oder PJ erreichen zumeist ein bereits vorselektiertes Kollektiv besonders Interessierter. Aussagen, die anhand dieser Gruppe getroffen werden, müssen entsprechend des Querschnitts der Studierenden eingeordnet werden. Ein nicht geringer Anteil Studierender erklärt sich jedoch in den Angeboten zur Stärkung der Rolle in Praktikum und Fachgebiet (rechtliche Grundlagen der Famulatur bzw. des PJ, Angebote zu MentorInnengesprächen der Fächer) als durchaus offen und dankbar für die Unterstützung durch fachspezifische „Insider“-Informationen. Vorarbeiten der AutorInnen konnten zeigen, dass dies insbesondere für die Chirurgie zu einer Verstärkung positiver Praktikumserlebnisse beiträgt [4].

Eine extrakurrikulare Veranstaltung erlaubt einerseits, die kurrikulare Lehre in ihrem aktuellen Umfang beizubehalten und andererseits, diese gleichzeitig zu beleben. Ein Ausbau extrakurrikularer Angebote allein wäre jedoch nicht zielführend. Vorhandene und bewährte Lehrveranstaltungen müssen bei allem Anspruch hinsichtlich Innovation und fakultativer Zusatzangebote effektiv genutzt und sinnvoll in eine abwechslungsreiche Lehrvielfalt integriert werden.

Während die dargestellten Konzepte vor allem an Universitäten praktikabel und umsetzbar erscheinen, wäre es sinnvoll, sich an nichtuniversitären Kliniken konsequent an Vorgaben und Erfahrungen der Herkunftsfakultäten zu orientieren und eigene abgewandelte, vor allem praktisch realisierbare Modifikationen (z. B. im 1:1-Teaching einer/s individuellen FamulantInnen‑/PJ-lerInnen-Betreuerin/s oder Kleingruppenarbeit bei strukturierter/m Famulatur/Tertial und definierten Lernzielen) zu implementieren.

## Fazit

Durch die Verbesserung der Ausbildung in den praktischen Phasen des Humanmedizinstudiums ist neben einer mittelbaren Verbesserung der Lehre eine Stärkung des Interesses am Fach und damit der Nachhaltigkeit im Sinne eines „recruitments“ neuer Fachkräfte zu erwarten. Besonders innerhalb der Praxisphasen kann die Begeisterung der Studierenden für ein Fach erfolgen und ausgebaut werden. Hier sind entsprechende Effekte frühzeitiger Ansätze beschrieben [4, 8]. Es ist daher wichtig, dass sich die *Chirurgie in der Lehre anhaltend über den gesamten klinischen Teil der Ausbildung* engagiert. Während dies in der theoretischen Lehre strukturiert umgesetzt wird, bestehen weiterhin viele nutzbare Chancen in der Zeit der Praxisphasen, die Begeisterung für das Fach auf die Studierenden zu übertragen. In Praxisphasen und besonders durch vorbereitende Seminare *besteht durch ein enges Ausbildungsverhältnis zur/m betreuenden Stations‑, Fach- oder Oberärztin/-arzt und Kleingruppenarbeit* eine effektive und intensive Ausbildungsmöglichkeit, wenn es für die Studierenden darum geht, den Transfer theoretischen Wissens in praktisch-ärztliche Tätigkeit zu realisieren. Durch die Famulaturen ist eine frühzeitige Einflussnahme möglich, die insbesondere für die Chirurgie von größerer Bedeutung für u. a. die Nachwuchsrekrutierung ist [4]. Darüber hinaus jedoch bedürfen Praxiserfahrungen innerhalb des Kurrikulums eines inhaltlichen Anschlusses, um positive Effekte für das Fach einzuordnen und gezielt zu vertiefen. Das Chirurgietertial im PJ ist häufig die letzte Möglichkeit, die Medizinstudierenden für die Chirurgie zu gewinnen. Das positive Erleben des PJ-Tertials wird an die nachfolgenden Jahrgänge weitergetragen. Daher ist es wichtig,

- das Tertial zu strukturieren,
- die Lernziele zu definieren,
- PJ-MentorInnen zu benennen (und)
- die PJ-Studierenden angemessen zu begleiten (sowie)
- adäquates Feedback zu geben.

Die Vorbereitungsseminare zur Versorgungskompetenz nach dem Magdeburger Modell bereiten die Studierenden, wenn es um den *alltäglichen Auftrag einer Versorgung von anvertrauten PatientInnen* geht, realitätsnah auf ihre Praxisphasen vor. Darüber hinaus leisten sie einen Beitrag zur Stärkung der *interdisziplinären und -professionellen Zusammenarbeit* der Dozierenden. Der Austausch der unterschiedlichen Fachrichtungen und Professionen bietet neue Möglichkeiten der Zusammenarbeit und Verzahnung für den Berufsalltag. Diese Kooperation kommt nicht nur der PatientInnenversorgung zugute, sondern eröffnet auch die Möglichkeit zum weiterführenden Ausbau bzw. der Optimierung bestehender interdisziplinärer Lehrkonzepte.

## Footnotes

1 In diesem Text wird der Einfachheit halber nur die männliche Form verwendet. Die weibliche Form ist selbstverständlich immer mit eingeschlossen.

2 Das in 6 Abschnitte (Einführung, Basisteil, Anforderungskatalog, ausgesuchte chirurgische Krankheitsbilder und Operationen sowie Informationen zu PJ-Veranstaltungen und eine Anleitung zur Herstellung eines Fallberichtes) unterteilte einrichtungsspezifische Logbuch vermittelt in seiner klinischen, sowohl kasus- und diagnose- als auch interventions- bzw. operationsbezogenen Ausrichtung insbesondere Inhalte als Basis institutionell eruierter Lehrerfahrungen.
