# Erratum zu: Innovative fakultative Seminarkonzepte besonders klinisch-praktisch ausgerichteter Lehre zur Famulatur- und PJ-Vorbereitung aus spezifisch chirurgischer Sicht

**DOI:** 10.1007/s00104-022-01791-9

**Published:** 2023-03-30

**Authors:** Philipp Stieger, Claus Schildberg, Marc Gottschalk, Katrin Werwick, Jonathan Hunger, Felix Walcher, Frank Meyer, Christian Albert, Rüdiger C. Braun-Dullaeus

**Affiliations:** 1 Universitätsklinik für Kardiologie und Angiologie, Universitätsklinikum Magdeburg A. ö. R., Magdeburg, Deutschland; 2 Universitätsklinik für Allgemein- und Viszeralchirurgie, Universitätsklinikum der MHB im Verbund Brandenburg an der Havel, Brandenburg an der Havel, Deutschland; 3 Studiendekanat der Medizinischen Fakultät, Otto-von-Guericke-Universität zu Magdeburg, Magdeburg, Deutschland; 4 Klinik für Neurologie, Klinikum Magdeburg GmbH, Magdeburg, Deutschland; 5 Universitätsklinik für Unfallchirurgie, Universitätsklinikum Magdeburg A. ö. R., Magdeburg, Deutschland; 6 Universitätsklinik für Allgemein‑, Viszeral‑, Gefäß- und Transplantationschirurgie, Universitätsklinikum Magdeburg A. ö. R., Leipziger Str. 44, 39120 Magdeburg, Deutschland

**Erratum zu:**

**Chirurgie 2022**

10.1007/s00104-022-01757-x

In diesem Artikel fehlten die Adressdaten bzw. die Daten zur institutionellen Zugehörigkeit für die Autoren Marc Gottschalk, Claus Schildberg, Katrin Werwick, Jonathan Hunger, Felix Walcher, Rüdiger Braun-Dullaeus, Christian Albert und Philipp Stieger. Außerdem wurden die Autoren in falscher Reihenfolge aufgeführt, die Reihenfolge wurde in der Autorezeile und im Interessenskonflikt angepasst.

Der Hinweis zu den gleichberechtigten Autoren wurde infolgedessen ebenfalls korrigiert und lautet nun: „Die Autoren Philipp Stieger und Claus Schildberg sind gleichberechtigte Erstautoren.“

Desweiteren wurde der Vorname von Katrin Werwick fälschlicherweise als Kathrin angegeben.

Der Originalbeitrag wurde korrigiert.

